# Preoperative Prognostic Nutritional Index is a Significant Predictor of Survival with Bladder Cancer after Radical Cystectomy: a retrospective study

**DOI:** 10.1186/s12885-017-3372-8

**Published:** 2017-06-02

**Authors:** Ding Peng, Yan-qing Gong, Han Hao, Zhi-song He, Xue-song Li, Cui-jian Zhang, Li-qun Zhou

**Affiliations:** 1 Department of Urology, Peking University First Hospital, No. 8, Xishiku Street, Xicheng District, Beijing, 100034 China; 2 Institute of Urology, Peking University, Beijing, 100034 China; 3 National Urological Cancer Center, Beijing, 100034 China; 4 Urogenital Diseases (male) Molecular Diagnosis and Treatment Center, Peking University, Beijing, 100034 China

**Keywords:** Prognostic nutritional index, Bladder cancer, Radical cystectomy, Outcomes

## Abstract

**Background:**

To explore the prognostic significance of preoperative prognostic nutritional index (PNI) in bladder cancer after radical cystectomy and compare the prognostic ability of inflammation-based indices.

**Methods:**

We retrospectively analyzed data for 516 patients with bladder cancer who underwent radical cystectomy in our institution between 2006 to 2012. Clinicopathologic characteristics and inflammation-based indices (PNI, neutrophil/lymphocyte ratio [NLR], platelet/lymphocyte ratio [PLR], lymphocyte/monocyte ratio [LMR]) were evaluated by pre-treatment measurements. Overall survival (OS) and progression-free survival (PFS) were estimated by the Kaplan–Meier method and compared by log-rank test. Multivariate analysis with a Cox proportional hazards model was used to confirm predictors identified on univariate analysis. The association between clinicopathological characteristics and PNI or NLR was tested.

**Results:**

Among the 516 patients, the median follow-up was 37 months (interquartile range 20 to 56). On multivariate analysis, PNI and NLR independently predicted OS (PNI: hazard ratio [HR] = 1.668, 95% CI: 1.147–2.425, *P* = 0.007; NLR: HR = 1.416, 95% CI:1.094–2.016, *P* = 0.0149) and PFS (PNI: HR = 1.680, 95% CI:1.092–2.005, *P* = 0.015; NLR: HR = 1.550, 95% CI:1.140–2.388, *P* = 0.008). Low PNI predicted worse OS for all pathological stages and PFS for T1 and T2 stages. Low PNI was associated with older age (>65 years), muscle-invasive bladder cancer, high American Society of Anesthesiologists grade and anemia.

**Conclusion:**

PNI and NLR were independent predictors of OS and PFS for patients with bladder cancer after radical cystectomy and PNI might be a novel reliable biomarker for bladder cancer.

## Background

Radical cystectomy is the standard treatment for localized muscle-invasive bladder cancer (MIBC) and non-muscle invasive bladder cancer (NMIBC) unresponsive to intravesical therapy [1, 2]. Despite the advances in surgical skills and chemotherapy, the 5-year survival with all bladder cancer is 77.9% and only 33.0% and 5.4% for regional and distant disease [3]. Therefore, prognostic factors for bladder cancer are needed for treatment decision making and postoperative monitoring.

Several preoperative hematological parameters have been reported as prognostic biomarkers for bladder cancer. Prognostic indicators suggested have been based on albumin and C-reactive protein levels and platelet and blood count, such as neutrophil/lymphocyte ratio [NLR], platelet/lymphocyte ratio (PLR) and lymphocyte/monocyte ratio (LMR) [4–8]. In addition, prognostic nutritional index (PNI), which combines nutrition and inflammation status, has been found to predict outcomes in various cancers [9–14]. However, no study has evaluated the prognostic value of PNI in bladder cancer.

This study aimed to explore the prognostic significance of preoperative PNI in bladder cancer patients after radical cystectomy and compare the prognostic ability of inflammation-based indices.

## Methods

We retrospectively reviewed the medical data for 571 consecutive bladder cancer patients who underwent radical cystectomy between 2006 and 2012 in Peking University First Hospital. We excluded 55 patients with non-bladder cancer, were lost to follow-up or had a history of disease that could affect blood cell lines. Therefore, we analyzed data for 516 patients. Clinicopathological data including gender, age, smoking status, history of Diabetes Mellitus, hypertension, heart and cerebrovascular disease, histology type, operation style (open or laparoscopic), American Society of Anesthesiologists (ASA) grade, postoperative complications (including prolonged ileus, fever, wound infection, wound dehiscence, gastrointestinal bleeding, cardiac arrhythmia, myocardial infarction, urinary leakage, pneumonia and death), pathological lymph-node status, pathological T stage and differential grade were obtained from the medical database. Histological subtype was diagnosed by at least 2 experienced pathologists on the basis of the 1973 WHO criteria, and TNM staging was assessed by the American Joint Committee on Cancer cancer staging system (7th edition, 2010). Hematological factors including preoperative hemoglobin and albumin levels and complete blood counts were collected within 3 days before surgery. This study was approved by the Institutional Review Board of Peking University First Hospital.

### Statistical analysis

The endpoint of the study was overall survival (OS), calculated from the day of surgery to the time of all-caused death, and progression-free survival (PFS), as the period from the date of surgery to the time of disease recurrence, metastasis or death. All continuous data are shown as median (interquartile range [IQR]). PNI was calculated as albumin level (g/L) + 5 × lymphocyte count (10^9^/L), PLR as platelet/lymphocyte ratio, NLR as neutrophil/lymphocyte count, and LMR as lymphocyte/monocyte count. Receiver operating characteristic (ROC) curve analysis was used to compare the prognostic ability of each indicator for each OS and PFS event according to the area under the ROC curve (AUC) and to determine the best cutoff points. For each prognostic factor, patients were divided into 2 groups according to cutoffs. The Kaplan–Meier survival method were used to draw OS and PFS curves. Univariate analysis involved the log-rank test. Factors significant on univariate analysis were included in Cox proportional-hazards multivariate models, estimating hazard ratios (HRs) and 95% CIs. The association of clinicopathological characteristics and PNI or NLR was tested by Mann–Whitney U-test. Statistical significance was considered with two-sided *p* < 0.05. All statistical analyses involved use of SPSS v21.0 (IBM Inc. Chicago, IL, USA).

## Results

A total of 516 patients (median age 66 years, IQR 57–73; 80 females [15.5%]) were included in this study. The median follow-up was 37 months (IQR 20–56). At the end of follow-up, 164 (31.8%) patients had died from any cause and 188 (36.4%) showed disease progression. The clinicopathological characteristics of all patients are shown in Table 1. The tumor stage of all patients was T1 for 162 (31.4%), T2 for 161 (31.2%), T3 for 105 (20.3%), and T4 for 88 (17.1%). The 3- and 5-year OS was 75.3% and 69% and PFS was 63.7% and 59.7%. Median NLR was 2.34 (IQR 1.74–3.49), PLR: 133.8 (98.22–180.22), LMR: 4.37 (3.30–5.72), PNI: 47.8 (44.66–51.58).

**Table 1:** Baseline clinicopathological characteristics of patients with bladder cancer

Characteristics	Total *n* = 516
Age, years, median (IQR)	66 (35–91)
Female sex, n (%)	80 (15.5%)
Histology type, n (%)	
UC	488 (94.6%)
NUC	28 (5.4%)
Pathological grade, n (%)	
2	131 (25.4%)
3	385 (74.6%)
Smoking history	161 (31.2%)
Diabetes Mellitus	56 (10.9%)
Hypertension	149 (28.9%)
Heart disease	55 (10.7%)
Cerebrovascular disease	17 (3.3%)
pT stage, n (%)	
1	162 (31.4%)
2	161 (31.2%)
3	105 (20.3%)
4	88 (17.1%)
pN status, n (%)	
negative	81 (15.7%)
positive	435 (84.3%)
ASA grade, n (%)	
1&2	436 (84.5%)
3&4	80 (15.5%)
Surgery style	
open	409 (79.3%)
laparoscopic	107 (20.7%)
Postoperative complications	
present	73 (8.3%)
absent	443 (91.7%)
Anemia	
present	144 (27.9%)
absent	372 (72.1%)
Hypoalbuminemia	
present	74 (14.3%)
absent	442 (85.7%)
NLR, median (IQR)	2.34 (1.74–3.49)
PLR, median (IQR)	133.8 (98.22–180.22)
LMR, median (IQR)	4.37 (3.30–5.72)
PNI, median (IQR)	47.8 (44.66–51.58)

*UC* urothelial carcinoma, *NUC* non-urothelial carcinoma, *ASA* American Society of Anesthesiologists, *PNI* prognostic nutritional index, *NLR* neutrophil-lymphocyte ratio, *PLR* platelet-lymphocyte ratio, *LMR* lymphocyte-monocyte ratio, *IQR*, interquartile range

**Fig. 1:**
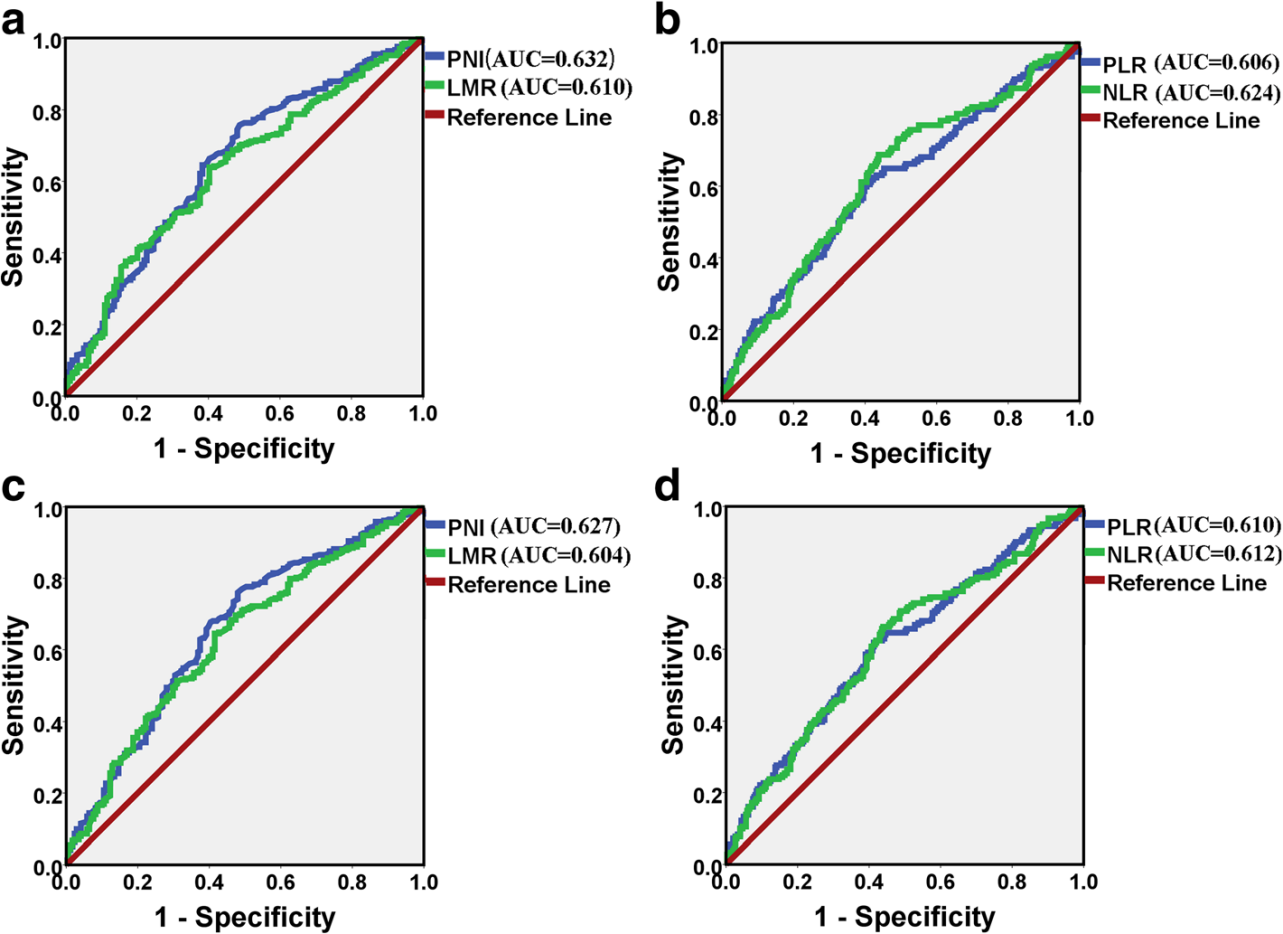
Receiver operating characteristic (ROC) curves for overall survival **a**, **b** and progression-free survival **c**, **d** for PNI,LMR,PLR and NLR

The AUC value was greater for PNI than the other 3 factors for estimating OS and PFS (Fig. 1). We determined the cutoff values for the 4 factors for OS and PFS by calculating the maximum Youden index (OS: PNI-46.025, NLR-2.303, PLR-136.125, LMR-4.099; PFS: PNI-47.20, NLR-2.288, PLR-135.247, LMR-4.099). Then patients were divided into low- and high-risk groups according to the ratios.

**Fig. 2:**
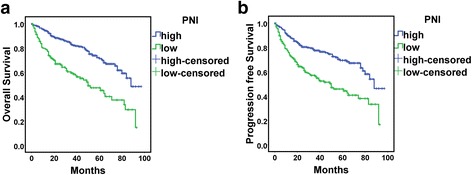
Kaplan-Meier survival curves for overall survival **a** and progression-free survival **b** for PNI

On univariate analysis, significant indicators for both OS and PFS were older age (>65 years), high tumor grade, pT2 or greater, positive lymph node status, history of heart and cerebrovascular disease, high ASA grade, hypoalbuminemia, anemia, postoperative complications and the 4 indicators (PNI, PLR, NLR, LMR) (Table 2). As compared with high PNI, low PNI was associated with worse OS and PFS (Fig. 2).

**Table 2:** Univariate and multivariate analyses of prognostic factors for overall survival and progression-free survival

Variable	Univariate	Multivariate	
	*P* value	HR (95% CI)	*P* Value
Overall survival			
Age (>65 vs. ≤65)	**<0.001**	1.615 (1.116–2.337)	**0.011**
Gender (male vs. female)	0.264		
Histology type (NUC vs. UC)	0.363		
Pathological grade (3 vs. 2)	**<0.001**		
pT (pT2 or greater vs. pT1)	**<0.001**	2.796 (1.700–4.598)	**<0.001**
pN status (positive vs. negative)	**<0.001**	1.682 (1.141–2.480)	**0.009**
Smoking history	0.937		
Hypertension	0.480		
Diabetes Mellitus	0.534		
Heart disease	**0.018**		
Cerebrovascular disease	**0.032**		
ASA grade (3&4 vs. 1&2)	**<0.001**	1.641 (1.113–2.418)	**0.012**
Hypoalbuminemia	**<0.001**		
Anemia	**<0.001**		
Postoperative complications	**0.003**	1.607 (1.076–2.400)	**0.020**
PNI (low vs. high)	**<0.001**	1.668 (1.147–2.425)	**0.007**
PLR (high vs. low)	**<0.001**		
NLR (high vs. low)	**<0.001**	1.416 (1.094–2.016)	**0.014**
LMR (low vs. high)	**<0.001**		
Progression-free survival			
Age (>65 vs. ≤65)	**<0.001**		
Gender (male vs. female)	0.304		
Histology type (NUC vs. UC)	0.683		
Pathological grade (3 vs. 2)	**<0.001**		
pT (pT2 or greater vs. pT1)	**<0.001**	2.560 (1.677–3.906)	**<0.001**
pN status (positive vs. negative)	**<0.001**	1.871 (1.306–2.680)	**0.001**
Smoking history	0.952		
Hypertension	0.604		
Diabetes Mellitus	0.681		
Heart disease	**0.012**		
Cerebrovascular disease	**0.033**		
ASA grade (3&4 vs. 1&2)	**<0.001**	1.561 (1.086–2.243)	**0.016**
Hypoalbuminemia	**<0.001**		
Anemia	**<0.001**		
Postoperative complications	**0.004**		
PNI (low vs. high)	**<0.001**	1.680 (1.092–2.005)	**0.011**
PLR (high vs. low)	**<0.001**		
NLR (high vs. low)	**<0.001**	1.550 (1.140–2.388)	**0.008**
LMR (low vs. high)	**<0.001**		

*UC* urothelial carcinoma, *NUC* non-urothelial carcinoma, *ASA* American Society of Anesthesiologists, *PNI* prognostic nutritional index, *NLR* neutrophil-lymphocyte ratio, *PLR* platelet-lymphocyte ratio, *LMR* lymphocyte-monocyte ratio

Significant values are in bold

Thus, these variables were included in a Cox proportional-hazards model. Independent risk factors for OS were older age (>65 years; HR = 1.615, 95% CI:1.116–2.337, *P* = 0.011), pT2 or greater (HR = 2.796, 95% CI:1.700–4.598, *P* < 0.001), positive lymph node status (HR = 1.682, 95% CI:1.141–2.480, *P* = 0.009), high ASA grade (HR = 1.641, 95% CI:1.113–2.418, *P* = 0.012), postoperative complications (HR = 1.607, 95% CI: 1.076–2.400, *P* = 0.020),low PNI (HR = 1.668, 95% CI: 1.147–2.425, *P* = 0.007) and high NLR (HR = 1.416, 95% CI:1.094–2.016, *P* = 0.0149). For PFS, independent risk factors were pT2 or greater (HR = 2.560, 95% CI:1.677–3.906, *P* < 0.001), positive lymph node status (HR = 1.871, 95% CI:1.306–2.680, *P* = 0.001), high ASA grade (HR = 1.561, 95% CI:1.086–2.243, *P* = 0.016), low PNI (HR = 1.680, 95% CI:1.092–2.005, *P* = 0.015) and high NLR (HR = 1.550, 95% CI:1.140–2.388, *P* = 0.008).

**Fig. 3:**
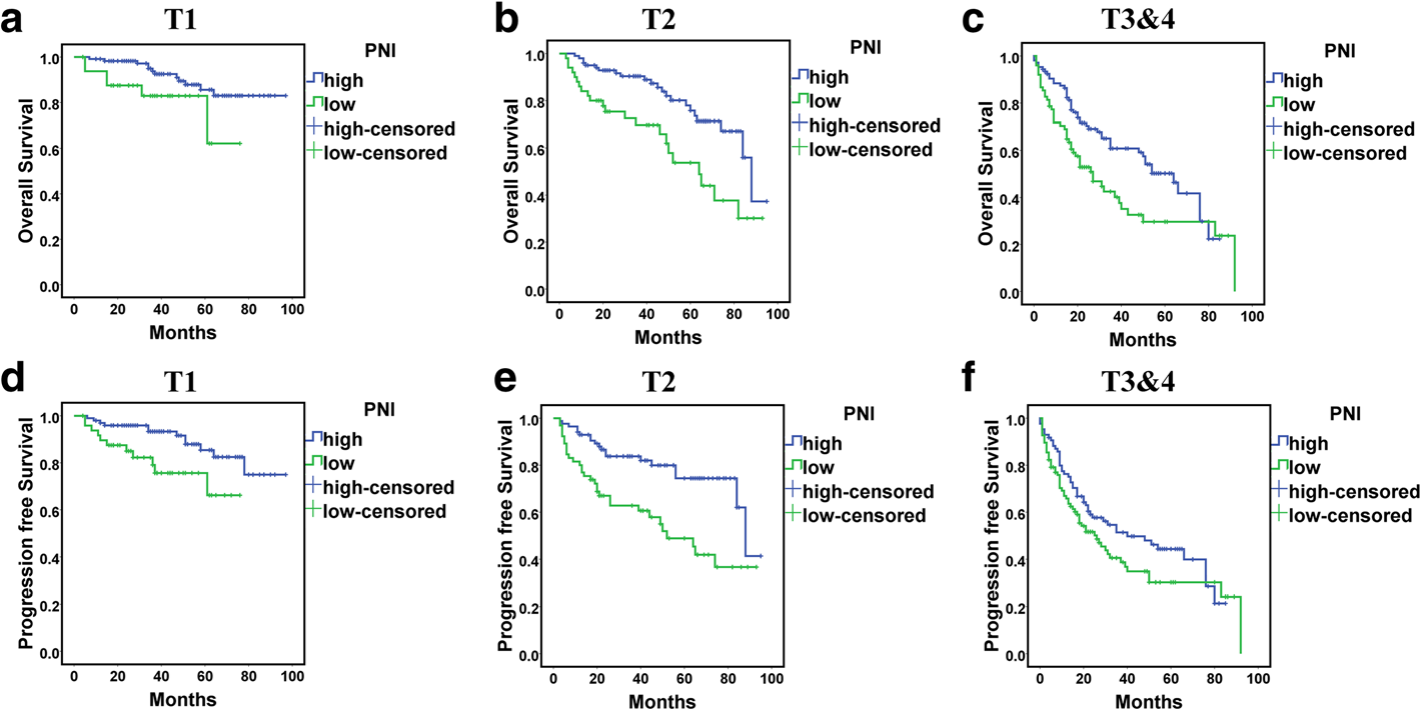
Kaplan-Meier survival curves for overall survival a-c and progression-free survival (D-F) for PNI at different tumor stages

Inflammatory status may be affected by disease stage. Therefore, we classified patients into 3 groups by pathological stage (Fig. 3). OS was shorter for patients with low than high PNI with all stages (stage 1: *P* = 0.042, stage 2: *P* = 0.002, stages 3 and 4: *P* = 0.012). However, PFS was shorter for patients with low PNI only with stage 1 or 2 disease (stage 1: *P* = 0.014, stage 2: *P* = 0.001, stages 3 and 4: *P* = 0.141).

We then assessed PNI and NLR for patients with different clinicopathological characteristics. Low PNI was associated with older age (>65 years), pT2 or greater, high ASA grade and anemia (Table 3). Meanwhile, elevated NLR level was associated with older age (>65 years), high grade and ASA grade, pT2 or greater, positive lymph node status, cerebrovascular disease and anemia.

**Table 3:** Relationship between clinicopathological characteristics and PNI or NLR

Variable	N	PNI	*P value*	NLR	*P value*
Gender			0.068		0.406
male	436	48.05 (44.95–51.75)		2.39 (1.77–3.47)	
female	80	46.90 (43.87–50.05)		2.21 (1.59–3.97)	
Age, years			**<0.001**		**<0.001**
> 65	278	46.92 (43.33–49.68)		2.61 (1.95–3.91)	
≤ 65	238	49.62 (46.25–53.22)		2.15 (1.68–2.92)	
Histology type			0.396		0.667
UC	488	47.80 (44.65–51.55)		2.33 (1.76–3.49)	
NUC	28	48.95 (46.15–53.05)		2.67 (1.66–3.54)	
Pathological grade			0.086		**0.048**
2	131	48.65 (45.25–52)		2.24 (1.70–2.90)	
3	385	47.65 (44.52–51.15)		2.43 (1.76–3.73)	
T stage			**<0.001**		**<0.001**
pT2 or greater	354	47.35 (44.05–50.65)		2.52 (1.90–3.93)	
pT1	162	49.00 (46.20–52.97)		2.06 (1.63–2.68)	
N status			0.250		**0.041**
negative	435	48.00 (44.90–51.65)		2.29 (1.71–3.42)	
positive	81	47.10 (43.87–51.02)		2.50 (1.99–3.96)	
Smoking			0.109		0.524
no	355	47.55 (44.40–51.57)		2.39 (1.72–3.64)	
yes	161	48.70 (45.42–51.60)		2.33 (1.81–3.17)	
Diabetes Mellitus			0.948		0.179
no	460	47.8 (44.75–51.60)		2.32 (1.73–3.44)	
yes	56	48 (44.50–51.28)		2.72 (1.81–3.99)	
Hypertension			0.862		0.067
no	367	47.80 (44.75–51.65)		2.27 (1.71–3.19)	
yes	149	48.02 (44.57–51.15)		2.50 (1.82–3.75)	
Heart disease			0.177		0.516
no	461	47.97 (44.90–51.65)		2.31 (1.73–3.53)	
yes	55	47.22 (43.70–50.56)		2.48 (1.78–3.09)	
Cerebrovascular disease			0.277		**0.045**
no	499	47.95 (44.71–51.6)		2.32 (1.72–3.44)	
yes	17	47.10 (42.77–49.10)		2.69 (2.22–4.04)	
Surgery style			0.278		0.806
open	409	47.95 (44.9051.75)		2.37 (1.73–3.65)	
laparoscopic	107	47.60 (44.2051.25)		2.28 (1.80–3.20)	
Postoperative complications			0.129		0.730
no	443	48.00 (45.05–51.55)		2.33 (1.76–3.47)	
yes	73	46.35 (43.75–51.78)		2.37 (1.59–3.61)	
ASA grade			**<0.001**		**0.031**
1&2	436	48.05 (45.25–51.90)		2.30 (1.725–3.21)	
3&4	80	46.00 (42.50–49.37)		2.53 (2.05–3.99)	
Anemia			**<0.001**		**<0.001**
no	372	49.07 (46.502.93)		2.23 (1.69–2.96)	
yes	144	44.55 (40.90–47.38)		3.01 (2.00–4.42)	

*UC* urothelial carcinoma, NUC non-urothelial carcinoma, *ASA* American Society of Anesthesiologists, *PNI* prognostic nutritional index, *NLR* neutrophil-lymphocyte ratio, *PLR* platelet-lymphocyte ratio, *LMR* lymphocyte-monocyte ratio

Significant values are in bold

## Discussion

Inflammation-based ratios are representative biomarkers of host inflammation response that predict the prognosis of cancer. In this study, we assessed the prognostic value of PNI and compared the prognostic ability of inflammation-based indices in bladder cancer patients who underwent radical cystectomy. By univariate and multivariate analyses, we found that PNI and NLR as prognostic and independent risk factors for both OS and PFS.

Immune cells play an important role in tumorigenesis, development and metastasis. Neutrophils can interact with tumor cells and secrete cytokines and chemokines which could promote tumor proliferation, angiogenesis and metastasis [15]. For example, neutrophils could secrete vascular endothelial growth factor (VEGF) into the circulation and VEGF is essential for tumor angiogenesis, metastasis and drug resistance. On the other hand, the role of lymphocytes is mainly through the tumor immune surveillance and tumor cell clearance to inhibit the tumorigenesis and development. In addition, neutrophils in the tumor microenvironment could also interact with lymphocytes and reduce the antitumor effects of activated T cells and natural killer (NK) cells [16, 17]. Therefore, an elevated NLR represent a neutrophilia and lymphocytopenia, which reflected the imbalance in the immune response. As a simple systemic inflammation response marker, NLR has been recommended associated with worse recurrence-free, disease-specific, and overall survival in patients with bladder cancer [18]. In the present study, NLR was independent risk factor for OS and PFS for bladder cancer patients and this is consistent with previous studies.

In addition to inflammation response, nutrition status is another important prognostic impact for cancer patients. Hypoalbuminemia has been demonstrated associated with cancer recurrence and decreased OS in bladder cancer patients after RC [19, 20]. Moreover, increasing evidence indicated that hypoalbuminemia in cancer patients is related with inflammatory imbalance as well as cancer cachexia. Lambert et al. [19] evaluated 187 bladder cancer patients and found 31(16.5%) patients were in the low-albumin cohort(albumin <3.5 g/dL). The OS was lower in low-albumin group than those with normal albumin and the complication rates were also higher in the group with low albumin. Djaladat et al. [20] reported 197 patients (13.4%) from a 1964 bladder cancer patients cohort had a low albumin level (<3.5 g/dL). In multivariable analysis for OS and RFS, low albumin remained independently associated with decreased OS and RFS. In our study, we observed 74 (13.66%) patients with albumin <3.5 g/dL and hypoalbuminemia was predictor for OS and PFS in univariate analysis. Those findings support the importance of albumin level in prognosis for bladder cancer.

PNI was first created by Onodera et al. to evaluate the inflammation and nutrition status of patients after gastrointestinal surgery [21]. Because PNI is calculated by serum albumin and lymphocyte count, decreased PNI represents hypoalbuminemia and decreased lymphocyte count, both responsible for worse outcomes in cancer patients. Since then, it has been a predictor of survival in several solid tumors including colorectal, breast, oesophageal, hepatocellular, renal and lung cancer [9–11, 13]. Ryuma Tokunaga et al. [22] compared the systemic inflmmatory and nutritional scores for colorectal cancer patients after curative resection and found PNI was a better predictive score than NLR, PLR and CRP.

Several studies also have compared inflammation-based ratios to find a good marker for bladder cancer and NLR or LMR was reported as the best [7, 23, 24]. These results differing from ours may be due to differences in patient characteristics and populations. In our study, NLR but not LMR remained an independent factor of survival on multivariate analysis, which may support the significance of NLR. In addition, these studies did not include PNI, a comprehensive and easily measured indicator. Therefore, we investigated PNI in the prognosis of bladder cancer and found it as a predictor of OS and PFS.

Our study has some limitations. First, this was a retrospective observational study and the inherent retrospective and nonrandomized nature may have led to selection bias. Second, we did not measure blood cell counts at regular intervals after radical cystectomy and could not explore the predictive value of the change in inflammation-based biomarkers pre- and post-radical cystectomy. Finally, this study was a single, tertiary-care institution study and our findings require well-controlled and multiple-institution studies for external validation.

## Conclusion

Both PNI and NLR are independent risk factors for OS and PFS. PNI may be an additional easily measured biomarker for stratifying risk preoperatively for bladder cancer patients who undergo radical cystectomy.

## Abbreviations

PNI: Prognostic nutritional index
OS: Overall survival
PFS: Progression free survival
MIBC: Muscle-invasive bladder cancer
NMIBC: Non-muscle invasive bladder cancer
IQR: Interquartile range
ASA: American Society of Anesthesiologists
ROC: Receiver operating characteristic
AUC: Area under the receiver operating characteristic curve
